# Specific Monoclonal Antibody Overcomes the *Salmonella enterica* Serovar Typhimurium’s Adaptive Mechanisms of Intramacrophage Survival and Replication

**DOI:** 10.1371/journal.pone.0151352

**Published:** 2016-03-17

**Authors:** Swarmistha Devi Aribam, Tomoyuki Harada, Marta Elsheimer-Matulova, Taketoshi Iwata, Katsushi Kanehira, Hirokazu Hikono, Hidenori Matsui, Yohsuke Ogawa, Yoshihiro Shimoji, Masahiro Eguchi

**Affiliations:** 1 National Institute of Animal Health, NARO, 3-1-5 Kannondai, Tsukuba, Ibaraki, 305–0856, Japan; 2 Kitasato Institute for Life Sciences and Graduate School of Infection Control Sciences, Kitasato University, 5-9-1 Shirokane, Minato-ku, Tokyo, 108–8641, Japan; Robert Koch-Institute, GERMANY

## Abstract

*Salmonella*-specific antibodies play an important role in host immunity; however, the mechanisms of *Salmonella* clearance by pathogen-specific antibodies remain to be completely elucidated since previous studies on antibody-mediated protection have yielded inconsistent results. These inconsistencies are at least partially attributable to the use of polyclonal antibodies against *Salmonella* antigens. Here, we developed a new monoclonal antibody (mAb)-449 and identified its related immunogen that protected BALB/c mice from infection with *Salmonella enterica* serovar Typhimurium. In addition, these data indicate that the mAb-449 immunogen is likely a major protective antigen. Using *in vitro* infection studies, we also analyzed the mechanism by which mAb-449 conferred host protection. Notably, macrophages infected with mAb-449-treated *S*. Typhimurium showed enhanced pathogen uptake compared to counterparts infected with control IgG-treated bacteria. Moreover, these macrophages produced elevated levels of pro-inflammatory cytokine TNFα and nitric oxide, indicating that mAb-449 enhanced macrophage activation. Finally, the number of intracellular bacteria in mAb-449-activated macrophages decreased considerably, while the opposite was found in IgG-treated controls. Based on these findings, we suggest that, although *S*. Typhimurium has the potential to survive and replicate within macrophages, host production of a specific antibody can effectively mediate macrophage activation for clearance of intracellular bacteria.

## Introduction

*Salmonella enterica* serovar Typhimurium (*S*. Typhimurium) is a facultative, gram-negative bacterium that causes zoonotic food-borne disease worldwide. *S*. Typhimurium causes a severe invasive disease in mice, resulting in bacterial dissemination within phagocytes of the spleen and liver [1]. The ability of *S*. Typhimurium to survive and replicate within macrophages plays a major role in its pathogenicity [1–4]. In the host, the innate and acquired immune systems are critical for controlling *Salmonella* infection [5–8]. Both cellular and humoral factors participate in the host defense against *Salmonella* [9–14]; however, the mechanisms of bacterial clearance via specific antibodies remain obscure despite detailed evidence for antibody-mediated protection in mouse models [7,9,10,13,15].

Studies using the passive transfer of anti-*Salmonella* serum to B cell-deficient mice and analysis of anti-*Salmonella* antibody production have clearly shown that antibodies are important for immune protection [7,10,13,16–18]. According to some reports, antibodies contribute to the immunological response by opsonizing extracellular *Salmonella*, which facilitates its phagocytic uptake [10,12,15,19]. However, the exact bactericidal mechanisms and an antigen are not known. In earlier studies with polyclonal antibodies against *Salmonella*, we reported that the antibody-dependent phagocytic uptake of *Salmonella* induces high-frequency apoptotic cell death that facilitates a *Salmonella*-specific T cell response [10,20]. Moreover, serum bactericidal assays revealed that anti-*Salmonella* antibodies contribute to immunity by promoting the complement-mediated killing of bacteria [21–24]. Most evidence on the activity of *Salmonella*-specific antibodies was derived from studies using immune sera or polyclonal antibodies from mice immunized with heat-killed or attenuated *Salmonella*, which might preclude a clear understanding of the mechanisms of antibody-dependent clearance.

In our study, we prepared hybridoma-derived mAb-449 and identified its related immunogen, which conferred protection against infection with virulent *S*. Typhimurium. In vitro functional analysis revealed that pre-treatment of *S*. Typhimurium with mAb-449 enhanced macrophage activation and bacterial uptake and clearance, suggesting a mechanism for antibody-mediated protection against *Salmonella* infection.

## Materials and Methods

### Ethics statement

Six-week old female BALB/c mice were purchased from SLC Japan, Inc. (Hamamatsu, Japan) and maintained in accordance with the National Institute of Animal Health research animal resource guidelines. Up to four mice were kept in each cage and housed in a temperature-regulated room and had free access to food and water. The handling of the animals in the study was performed under Fundamental Guidelines for Proper Conduct of Animal Experiment and Related Activities in Academic Research Institutions under the jurisdiction of the Ministry of Education, Culture, Sports, Science and Technology, Japan. The specific experiments were approved by and conducted according to the guidelines of the experimental animal ethics committees of the National Institute of Animal Health (NIAH), Japan (Project license 12–025,13–012, 12–106). All animal experiments were performed to ameliorate suffering according to the guidelines of the experimental animal ethics committees of the NIAH.

### Bacterial strains

The *S*. Typhimurium Χ3306 (LD_50_ in BALB/c: <10 CFU) [10] was used in this study. Bacteria were grown overnight in Luria Bertani (LB) medium at 37°C.

### Bacterial challenge

One day after administration of mAb-449 at various concentrations in 200 μL PBS or 200 μL immune serum, and 4 weeks after immunization with lipopolysaccharide (LPS), mice were challenged with bacteria at 10-fold the LD_50_ dose via intraperitoneal infection [7,10]. Mice administered PBS, control IgG, or control serum were used as controls. Challenged mice were monitored daily for their body weight loss and any signs of sickness. Mice that were in a moribund condition or had lost more than 30% of body weight were considered to have reached an experimental endpoint, and were humanely euthanized by carbon dioxide-bottled gas according to the guidelines of the experimental animal ethics committees of Fundamental Guidelines for Proper Conduct of Animal Experiment and Related Activities in Academic Research Institutions and the NIAH. The number of mice surviving after two weeks of daily observation was used to determine the relative degree of protection.

### Immunization

Immunization of mice with LPS was carried out twice by subcutaneous injection of 1.0–2.5 μg emulsified in Freund’s incomplete adjuvant (Difco, Franklin Lakes, NJ, USA) [25,26]. The immune serum and control serum were used 4 weeks after immunization of mice with LPS, or with adjuvant only.

### *In vitro* infection studies

The mouse macrophage-like cell line RAW264.7 was obtained from American Type Culture Collection. RAW264.7 cells (1 × 10^5^ cells/well) were infected with various multiplicities of infections (MOIs) of Χ3306 [27]. In some experiments, MOI 1 of Χ3306 were treated with various concentrations of mAb-449 or control IgG or with 5 μg/mL F(ab’)_2_ for 30 min at 37°C before adding to the cell culture [10,27]. After 1 h of infection with the pre-treated Χ3306, RAW264.7 cells were washed with PBS and incubated with medium containing 100 μg/mL gentamicin for 1 h at 37°C in a 5% CO_2_ incubator. The medium was then replaced with fresh medium containing 30 μg/mL gentamicin. The culture medium was collected for cytokine and nitric oxide production assays, and the cells were lysed in 1 mL 0.2% Triton X-100 in PBS for 5 min to enumerate bacteria on LB plates as previously described [10].

### Syk kinase assay

RAW264.7 cells (1 × 10^5^ cells/well) were infected with MOI 1 of *S*. Typhimurium pre-treated with 5 μg/mL mAb-449 as described above. Endogenous Syk kinase activity was analyzed using a PathScan^®^ phospho-Syk (panTyr) sandwich ELISA kit (Cell Signaling, Danvers, MA, USA) according to the manufacturer’s instructions.

### ELISA to test for TNFα production

TNFα production by RAW264.7 cells was measured using a mouse TNFα ELISA Ready-SET-Go!^®^ kit according to the manufacturer’s instructions (eBioscience, San Diego, CA, USA).

### Nitric oxide production assay

Macrophage nitric oxide production was determined by measuring the concentration of nitrite, a stable metabolic byproduct from the reaction of nitric oxide and oxygen with Griess reagent as previously described [10]. For this, 100 μL /well of sample was transferred to a 96-well plate and mixed with an equal volume of Griess reagent (50:50 mix of 1% sulfanilamide in 2.5% phosphoric acid and 0.1% *N*-1-naphthylethylenediamide hydrochloride in distilled water). After a 5-min incubation at room temperature, absorbance was measured at 595 nm, and nitrite concentration was determined from a sodium nitrite standard curve.

### Electron microscopy and immunoblotting analysis of the protective antigen

For electron microscopy analysis, the bacterial suspension was placed on collodion-coated copper grids (400 mesh, Nisshin EM, Tokyo, Japan) for 2 min. The grids were incubated with mAb-449 or immune serum followed by colloidal gold-conjugated goat anti-mouse immunoglobulin (10-nm diameter, BBI Solutions, Cardiff, UK) before imaging with a transmission electron microscope (H-7500, Hitachi, Tokyo, Japan).

LPS was isolated from heat-killed bacteria by hot phenol-water extraction [28], and approximately 5 μg of the isolate was separated by 12% SDS-PAGE (Bio-Rad, Hercules, CA, USA) and analyzed by a modified silver staining procedure [29] or used for immunoblot analysis. For the latter, LPS samples were transferred onto polyvinylidene difluoride (PVDF) membranes (Bio-Rad). For dot blot analysis, *S*. Typhimurium lysate was prepared by resuspending the bacterial pellet in PBS and sonicating on ice as previously described [30]. The sonicated samples were centrifuged, and the total protein content in the supernatant was determined by bicinchoninic acid protein assay kit (Pierce, Rockford, IL, USA). *S*. Typhimurium whole cell lysate before or after treatment with endotoxin removal resin (Promega, Madison, WI, USA) or LPS were spotted onto nitrocellulose membranes. The PVDF and nitrocellulose membranes were then blocked with Blocking One solution (Nacalai Tesque, Kyoto, Japan) followed by primary antibody incubation in mAb-449-producing hybridoma culture supernatant (~2 μg/mL) and incubation in peroxidase-conjugated rabbit anti-mouse IgG2a secondary antibody (1:2000; Invitrogen, Carlsbad, CA, USA). Reactive bands were detected with 3,3-diaminobenzidine tetrahydrochloride (Wako, Osaka, Japan) and hydrogen peroxide.

### Statistical analysis

Prism 6 (GraphPad, CA, USA) was used for the statistical analyses. The survival rates were analyzed using the Kaplan-Meier log-rank test. Statistical analyses were performed using two-way ANOVA or Student’s *t*-test to compare groups. Data are presented as the mean ± standard deviation (SD) for 3–6 samples per group.

## Results

### mAb-449 monoclonal antibody conferred protection against *S*. Typhimurium infection

Previous investigations demonstrated that pre-treating *S*. Typhimurium with anti-*Salmonella* IgG from immune serum enhanced bacterial uptake by macrophages and imparted a specific protective effect against *S*. Typhimurium infection in vivo [10]. As expected, sera from mice immunized with *Salmonella* UF20 (*aroA*^*-*^) exhibited a high antibody titer that enhanced bacterial uptake by mouse macrophage-like RAW264.7 cells and conferred protection against *S*. Typhimurium infection in BALB/c mice (S1A and S1B Fig). To generate a mAb that enhances macrophage bacterial uptake, we used a modified in vitro infection assay [31] and determined that treatment of the *S*. Typhimurium strain Χ3306 with mAb-449 promotes their phagocytosis by RAW264.7 cells (S1C Fig) [10]. Further characterization by antibody subtyping identified the mAb-449 isotype as IgG2a (S1D Fig).

**Fig 1:**
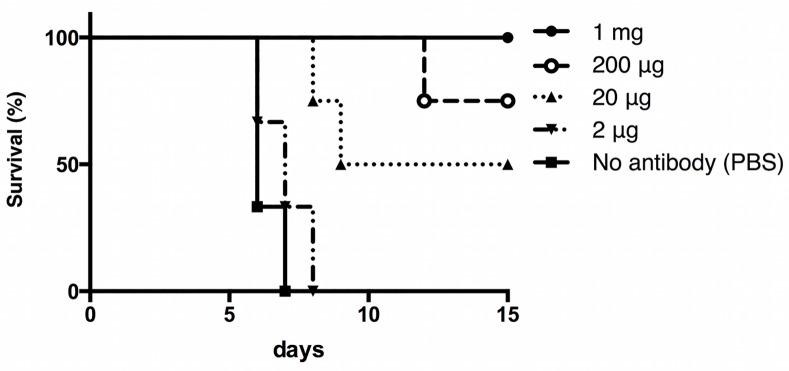
Immunoprotective potential of monoclonal antibody mAb-449 against *S*. Typhimurium infection. Mice were intravenously injected with mAb-449 1 day before intraperitoneal challenge with 10-fold the LD_50_ of *S*. Typhimurium (n = 4/group). Mice injected with PBS only were used as controls. The Kaplan-Meier log-rank test stratified by regimen was significant (*p* < 0.0001).

Subsequently, to determine whether the antibody could induce specific protective immunity to *Salmonella* infection in vivo, mAb-449 was administered to naïve mice at various concentrations the day before challenge with 10-fold the LD_50_ dose of *S*. Typhimurium (Fig 1). Notably, while PBS-administered control mice succumbed to disease within 7 days of *S*. Typhimurium challenge, mice inoculated with 1 mg or 200 μg mAb-449 survived. In addition, a lower dosage of 20 μg mAb-449 showed a protective effect in 50% of the population. Thus, these data indicate that mAb-449 administration confers a protective immunity against *S*. Typhimurium challenge.

### Functional analysis of mAb-449 antibody

**Fig 2:**
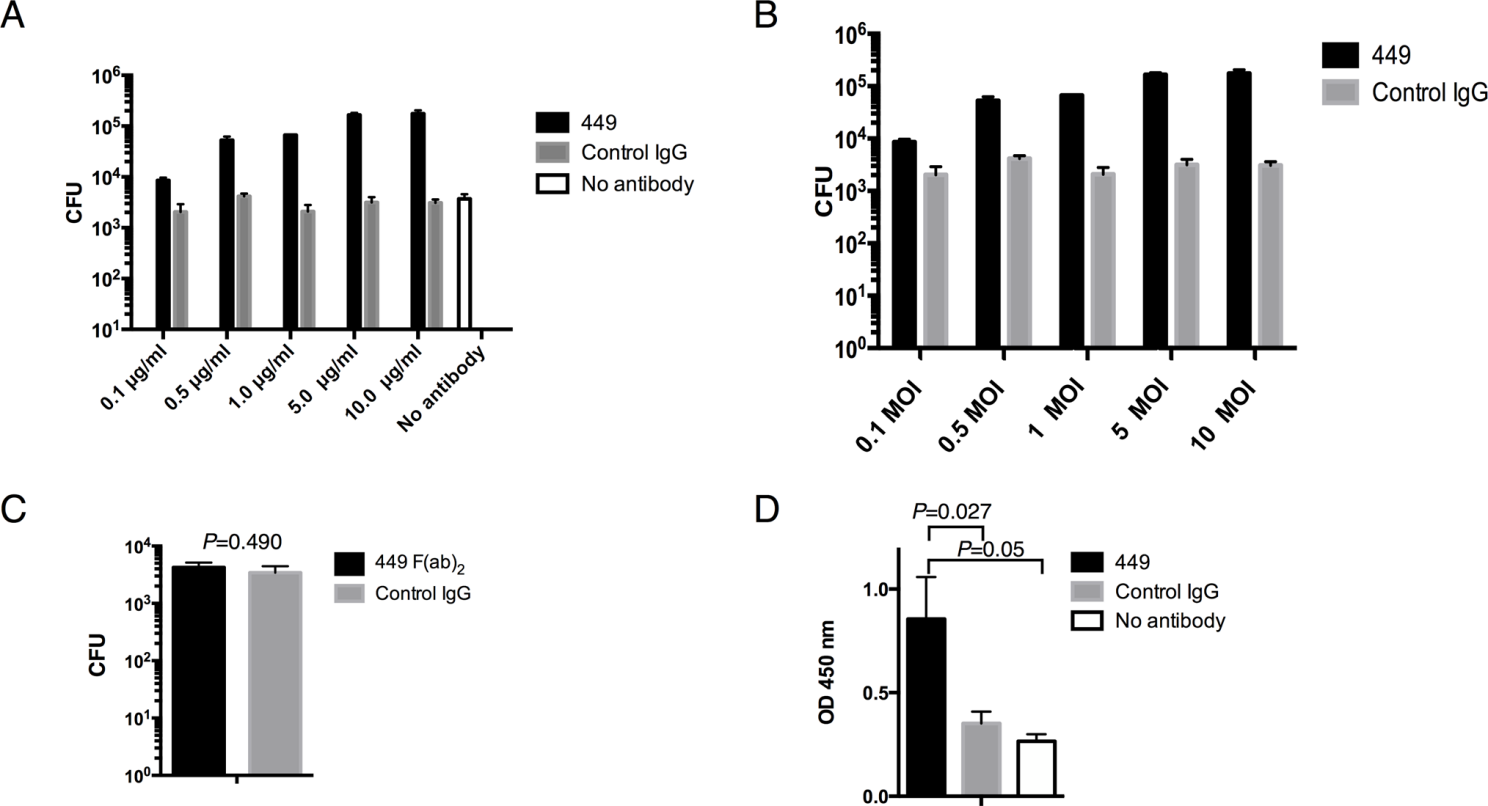
The effect of mAb-449 on bacterial uptake by RAW264.7 cells. Intracellular bacteria were quantified following infection at (A) MOI 1 of *S*. Typhimurium treated with 0.1, 0.5, 1, 5 or 10 μg/mL mAb-449, control IgG, or No-antibody (PBS); (B) MOI 0.1, 0.5, 1, 5, or 10 of *S*. Typhimurium treated with 5 μg/mL mAb-449 or control IgG; and (C) MOI 1 of *S*. Typhimurium treated with 5 μg/mL F(ab’)_2_ fraction of mAb-449 or control IgG. One hour after infection, intracellular bacteria were quantified by serial dilution plating on LB. (D) Syk kinase activation in *S*. Typhimurium-infected RAW264.7 cells was analyzed by ELISA. (A-D) Graphs show means and SD of triplicate readings. (A, B) Two-way ANOVA analyses showed significant differences in bacterial uptake with increased (A) mAb-449 concentration (*p* < 0.0001) or (B) MOI of mAb-449-treated *S*. Typhimurium (*p* < 0.0001). (C, D) Significance was assessed using Student’s t test.

Preliminary analyses demonstrated that mAb-449 could increase *S*. Typhimurium uptake by RAW264.7 cells (S1C Fig). Therefore, we performed in vitro infection assays and found significantly more intracellular bacteria following pre-treatment with mAb-449 than after pre-treatment with control IgG (Fig 2A). In addition, we also observed a dose-dependent increase in the number of adherent bacteria with increasing concentrations of mAb-449 (S2A Fig). Moreover, the number of adherent and intracellular bacteria also increased with higher MOIs (S2B–S2F Fig and Fig 2B). This finding was dependent on mAb-449 activity since treatment with mAb-449 induced significantly higher bacterial uptake than treatment with control IgG (Fig 2B). Furthermore, mAb-449 enhanced *S*. Typhimurium uptake by mouse macrophage-like J774.1 cells and peritoneal macrophages (S2G and S2H Fig).

Next, we investigated whether this bacterial uptake was mediated by the F(ab’)_2_ region of mAb-449. Treatment with only the F(ab’)_2_ region had no effect on *S*. Typhimurium uptake, indicating that the Fc moiety is required for the uptake of bacteria by macrophages (Fig 2C). Crosslinking of the macrophage Fc receptor (FcR) and the Fc component of IgG promotes phagocytosis by initiating intracellular signals conveyed by tyrosine kinase cascades [32]. To determine whether mAb-449 induced FcR-mediated signaling, phosphorylation of the tyrosine kinase Syk was examined in RAW264.7 cells following infection with *S*. Typhimurium (Fig 2D). Notably, the level of phospho-Syk was significantly higher in RAW264.7 cells infected with mAb-449-treated *S*. Typhimurium bacteria than in control IgG-treated or No-antibody (PBS)-treated *S*. Typhimurium. Altogether, these results suggest that mAb-449 functions by eliciting FcR activation on macrophages to enhance their uptake of opsonized bacteria.

### Activation of macrophages mediated by treatment with mAb-449

**Fig 3:**
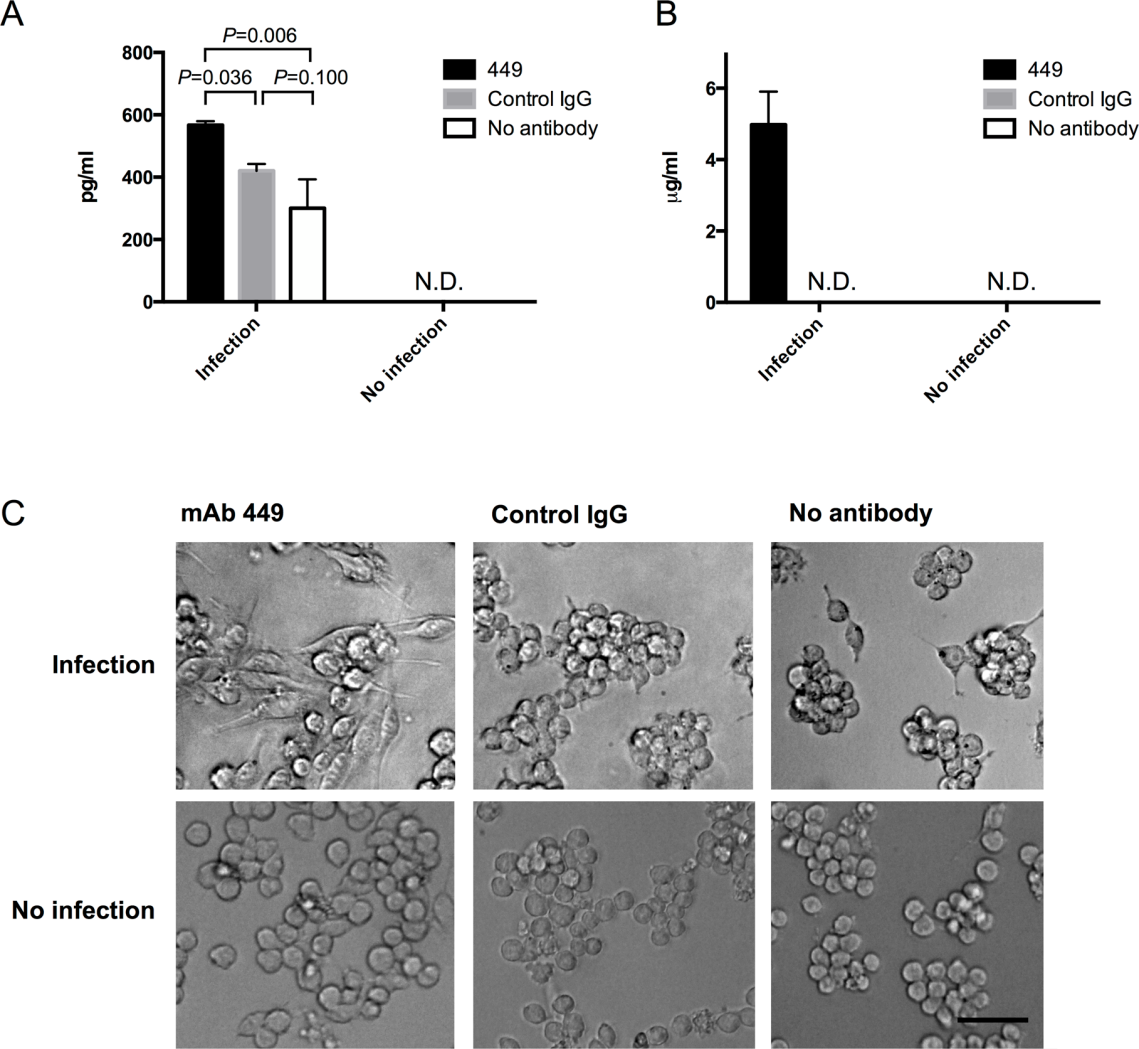
Role of mAb-449 in macrophage activation. RAW264.7 cells were infected with MOI 1 of *S*. Typhimurium or culture medium treated with mAb-449, control IgG, or No-antibody (PBS). (A) TNFα production by RAW264.7 cells 1 h after infection via ELISA. (B) Nitric oxide production was evaluated by Griess assay 36 h after infection. (A, B) Graphs show means and SD of triplicate readings. Significance was assessed using Student’s t test. N.D., not detected. (C) Image of RAW264.7 cells 36 h after infection with *S*. Typhimurium. Bar = 100 μm.

Since mAb-449 enhanced bacterial uptake by macrophages (Fig 2), we investigated whether macrophages could be activated by mAb-449. For this, we examined pro-inflammatory TNFα and nitric oxide production in the culture supernatants of infected RAW264.7 cells. A significant increase in secreted TNFα was detected in the supernatant of cells infected with mAb-449-treated *S*. Typhimurium compared to counterparts treated with control IgG or No-antibody (Fig 3A). Correspondingly, nitric oxide synthesis was markedly elevated in the supernatant of RAW264.7 cells infected with mAb-449-treated *S*. Typhimurium compared to supernatants from those infected with control IgG-treated or No-antibody-treated *S*. Typhimurium (Fig 3B). Furthermore, we inhibited the production of nitric oxide by using 100 μM *N*^G^-monomethyl-L-arginine (L-NMMA), a competitive inhibitor of nitric oxide synthase, in our in vitro experiment (S3 Fig). Cytopathology also revealed a notable difference in the morphology of RAW264.7 cells infected with mAb-449-treated *S*. Typhimurium compared to controls (Fig 3C). Furthermore, experiments using GFP-labeled *S*. Typhimurium indicated that mAb-449 enhanced uptake of *S*. Typhimurium into RAW264.7 cells (S4 Fig). Together, these data support a role of mAb-449 in triggering macrophage activation and phagocytic bacterial uptake.

### Intracellular bacterial clearance by activated macrophages

**Fig 4:**
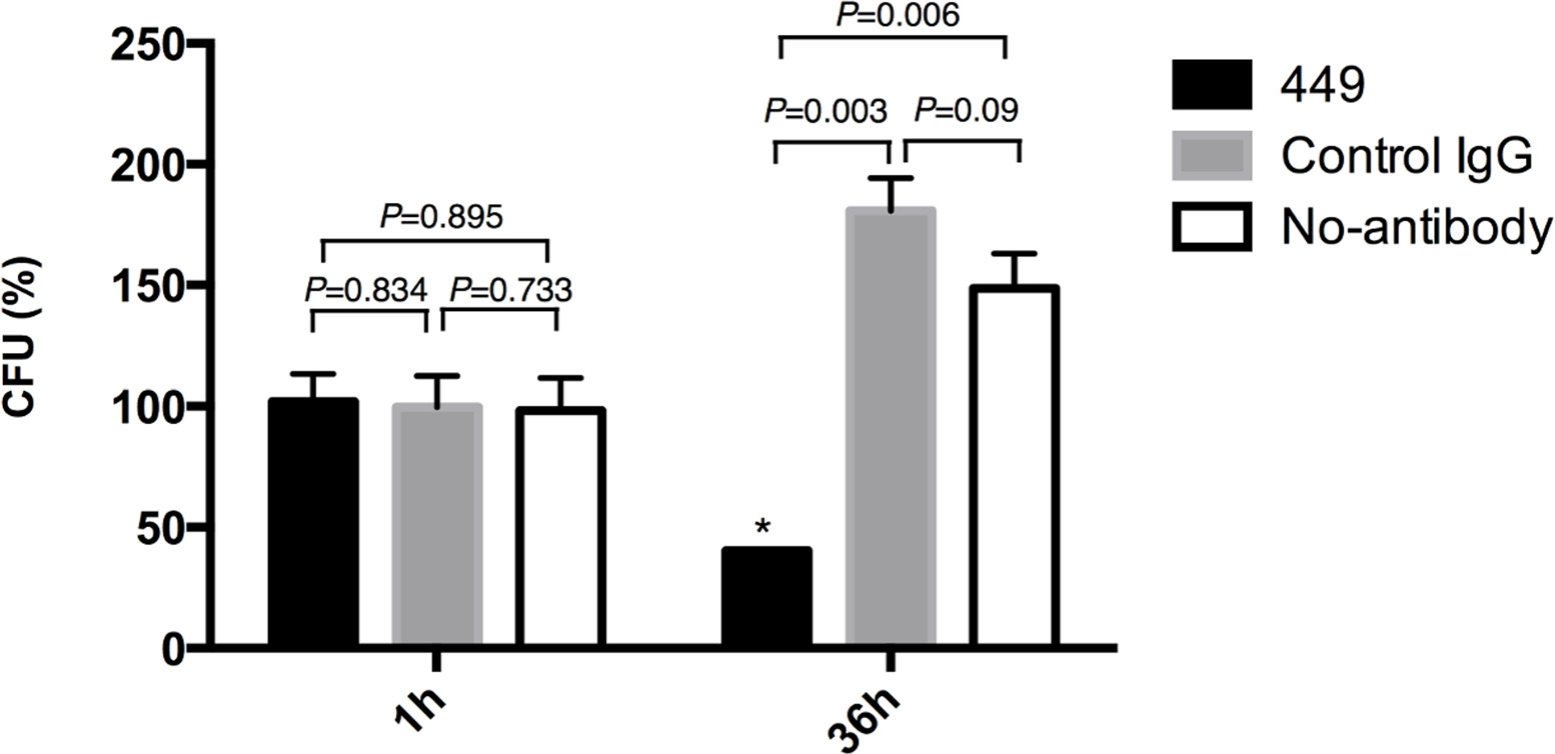
Effect of mAb-449 on intracellular bacterial killing. Relative CFU (%) of intracellular *S*. Typhimurium 36 h after infection in RAW264.7 cells compared to samples taken 1 h post infection. Graph shows means and SD of readings from two individual experiments performed in triplicates. Significance was assessed using Student’s t test. Asterisk indicates statistical significance when compared to no-inhibitor group (*p* < 0.01).

To analyze whether the survival and/or replication of mAb-449-treated bacteria is affected by macrophage activation, we enumerated intracellular bacteria with colony forming units (CFU) assay (S5 Fig). To facilitate the comparison of intracellular CFU for mAb-449-, control IgG-treated, and No-antibody *S*. Typhimurium, the CFU value for intracellular bacteria at 36 h post-infection was expressed as a percentage relative to that observed 1 h post-infection (Fig 4). These results indicated that the bactericidal activity of macrophages infected with mAb-449-treated S. Typhimurium is enhanced relative to negative control groups. Moreover, in similar numbers of intracellular bacteria with CFU as mAb-449-treated *S*. Typhimurium, that is MOI 10, the bactericidal activity of macrophages infected with mAb-449-treated *S*. Typhimurium is also enhanced relative to MOI 10 (S6 Fig). In addition, the nitric oxide synthesis in the supernatant of RAW264.7 cells infected with mAb-449-treated *S*. Typhimurium was also found to be higher than that from supernatants of RAW264.7 cells infected with MOI 10 of the bacteria (S7 Fig).

### Analysis of mAb-449 antigen specificity and protective immunity

**Fig 5:**
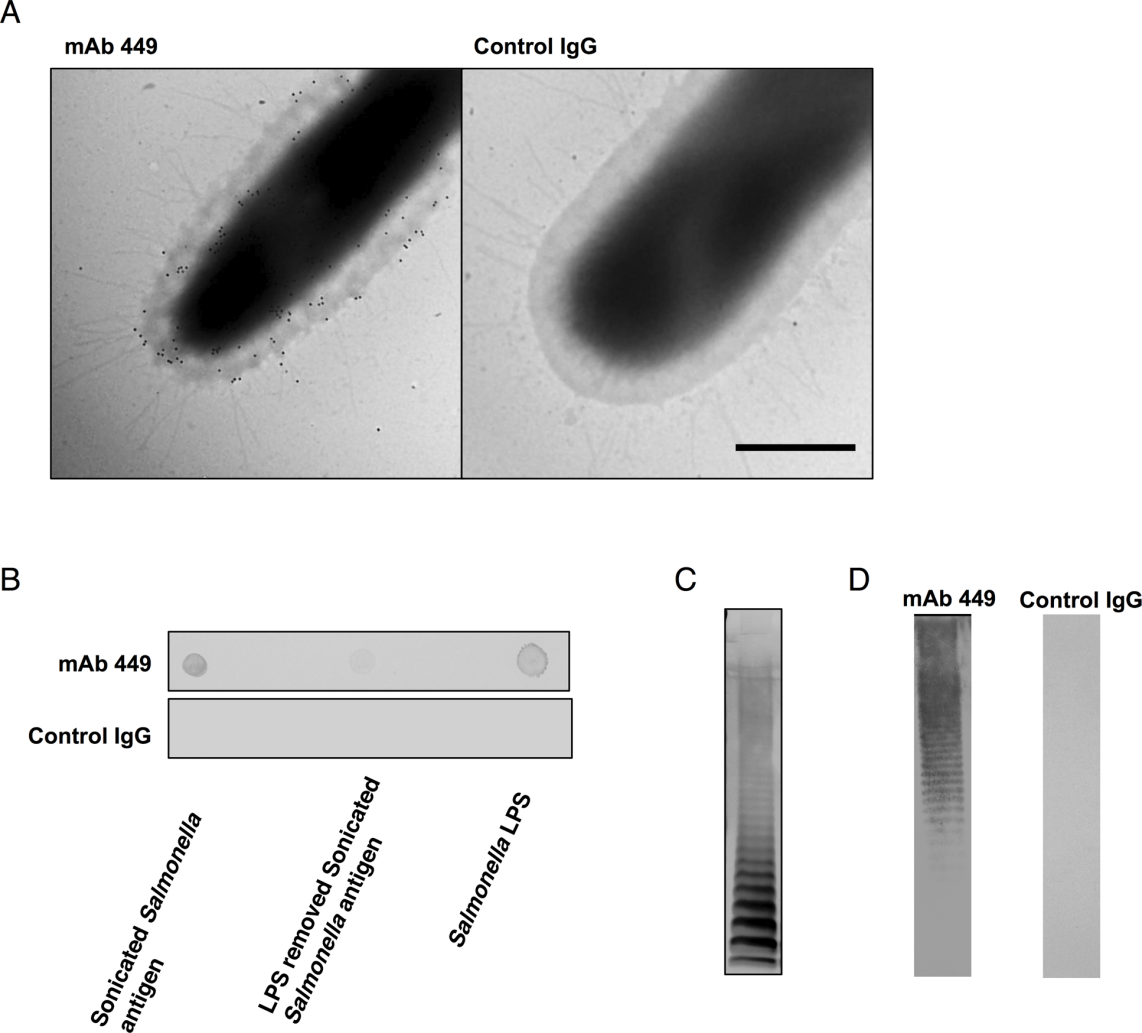
Antigen recognition by mAb-449 and its role in immunoprotection. (A) Electron microscopy images of mAb-449-treated *S*. Typhimurium. Black grains indicate mAb-449 binding regions on the bacterial cell surface. Bar = 500 nm. (B) Dot blot analysis showing antibody-antigen recognition with mAb-449 or control IgG and *S*. Typhimurium constituents using whole cell lysate, LPS-depleted lysate, and *S*. Typhimurium LPS. (C) *S*. Typhimurium LPS was analyzed by SDS-PAGE silver staining. (D) Immunoblotting to identify the antigenic constituent.

To identify the mAb-449 immunogen, we performed immunogold electron microscopy analysis and found that the antibody bound to the bacterial cell surface (Fig 5A). Since the outer membrane of gram-negative bacteria is often coated with LPS, we theorized that mAb-449 may specifically recognize this immunogen. Dot immunoblot analysis using *S*. Typhimurium whole cell lysate, LPS isolate, and LPS-depleted *S*. Typhimurium lysate revealed that mAb-449 bound to the whole cell lysate and LPS isolate but not LPS-depleted lysate (Fig 5B). Moreover, an immunoblot profile demonstrated that mAb-449 specifically recognizes the O-antigen region of LPS (Fig 5C and 5D and S8 Fig).

**Fig 6:**
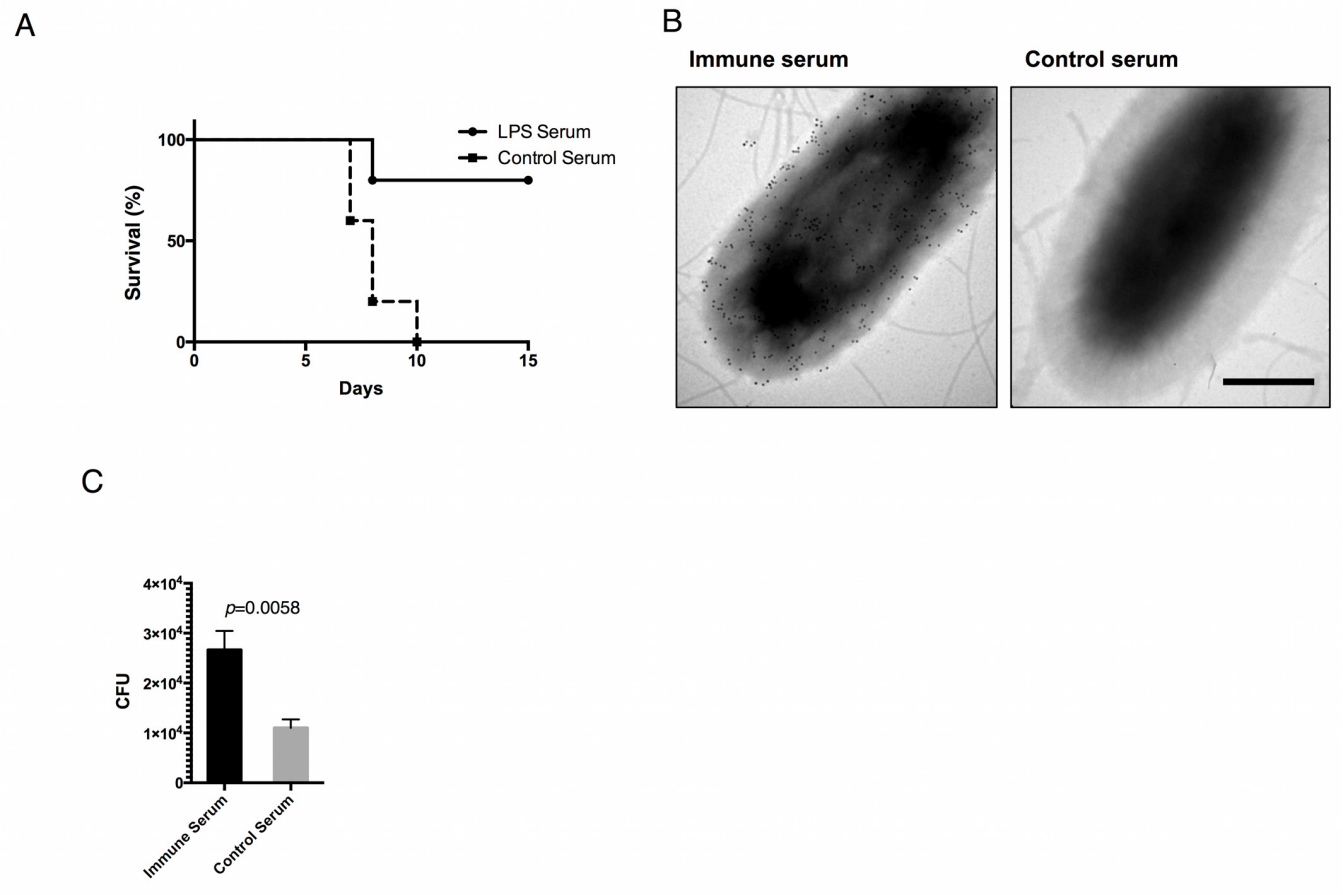
Immunoprotective potential of antigen of mAb-449 against *S*. Typhimurium infection. Mice transferred with anti-LPS serum were challenged with 10-fold the LD_50_ (n = 5/group). The Kaplan-Meier log-rank test stratified by regimen was significant (*p* = 0.01). (B) Immunogold electron microscopy analysis of *S*. Typhimurium. Black grains indicate the anti-LPS serum binding region on the bacterial cell surface. Bar = 500 nm. (C) Increased number of intracellular bacteria after infecting RAW267.4 cells with MOI 1 of *S*. Typhimurium treated with 1% anti-LPS serum or control serum. One hour after infection, intracellular bacteria were quantified by serial dilution plating on LB. Graphs show means and SD of triplicates. Significance was assessed using Student’s t test. * *P* = 0.0103.

To determine if mAb-449 specific antigen could confer protection from *S*. Typhimurium infection, mice were administered with anti-LPS serum and challenged with 10-fold the LD_50_ dose. As expected, the control serum administered mice died within 11 days of challenge, whereas more than 80% of anti-LPS serum administered mice survived for the 15 days they were monitored (Fig 6A). In addition, electron microscopy showed that anti-LPS serum detected the surface components of *S*. Typhimurium (Fig 6B). In vitro infection study also showed that anti-LPS serum treatment significantly enhanced bacterial uptake into macrophages (Fig 6C). These data indicate that mAb-449 antigen recognition generated a specific protective effect against *Salmonella* infection in vivo.

## Discussion

Several studies have shown that antibodies play a crucial role in mediating immunity against *Salmonella* infection; however, these studies are limited to the use of immune sera containing polyclonal antibodies [10,13,14,16,33]. Polyclonal antibodies consist of several immunoglobulins with varied stoichiometries, specificities, and characteristics, which can influence the efficacy of active antibodies and impede the mechanistic understanding of antibody function [34]. Therefore, in this study, we developed the mAb-449 to characterize immunoglobulin-mediated immunity against *S*. Typhimurium infection (Fig 1).

The protective efficacy of mAb-449 was associated with enhanced bacterial uptake by macrophages (Fig 2). Using RAW264.7 cells, we demonstrated that bacterial uptake is mediated by the interaction between mAb-449 and FcRs (Fig 2C and 2D). This suggests that circulating anti-LPS antibody may target extracellular bacteria during infection by FcR-mediated phagocytosis [12]. FcRs are essential for antibody-mediated protection, as FcR-knockout mice immunized with attenuated *S*. Typhimurium failed to clear a subsequent challenge infection [15].

FcR activation potentiates macrophage activation, which is characterized by bactericidal activity and the production of pro-inflammatory cytokines and reactive oxygen intermediates such as nitric oxide [35–37]. Accordingly, macrophages infected with mAb-449-treated *S*. Typhimurium exhibited elevated TNFα and nitric oxide levels and altered cellular morphology (Fig 3A and 3B). Most notably, the number of intracellular bacteria in the mAb-449-treated macrophages was significantly decreased (Fig 4), which is likely due to enhanced phagocytic killing. Furthermore, the potential of phagocytic killing was found to decrease when macrophages were treated with nitric oxide synthases inhibitor (S9 Fig). However, phagocytic killing was not completely suppressed by nitric oxide synthases inhibitor and we suggest that other active oxygen has an effect on phagocytic killing against *S*. Typhimurium [37]. From these results, we speculate that treatment with a specific antibody is sufficient to overcome the adaptive mechanisms utilized by *Salmonella* species to facilitate intramacrophage survival and replication [38,39]. Thus, the continued development of mAb-449 may provide more information on the protective role of antibodies against intracellular bacteria [40]. Because other intracellular pathogens such as *Listeria monocytogenes* and *Mycobacterium tuberculosis* have modes of infection similar to *Salmonella*, we believe that these analyses would also be relevant to such bacteria [40–42].

Importantly, our analyses revealed that mAb-449 specifically recognized LPS on the outer surface of *S*. Typhimurium bacteria (Fig 5) and likely binds the non-toxic O-antigen region (Fig 5C). Because LPS is toxic to the host, its use as a vaccine candidate is often limited; however, this toxicity is inhibited following alkali treatment, which yields D-LPS (O-antigen region) [42,43]. The ability of O-antigen region to elicit protective immunity suggests that it may be a potential candidate for vaccine development [26].

An important aspect of vaccine design is to identify a major, protective antigen that elicits the production of protective antibodies [34,44]. LPS is known to stimulate the cellular immune response upon recognition by Toll-like receptor 4 (TLR4) [45,46]; however, the mechanism of B cell-mediated immunity in response to LPS is not clearly understood [47]. Notably, our results demonstrated that inoculation with anti-LPS antibody serum confers specific protective immunity against *Salmonella* infection in mice by inducing macrophage activation and bactericidal activity (Fig 4), suggesting that the mAb-449 immunogen is likely a major protective antigen.

The mechanisms underlying the protective efficacy of antibodies specific to *Salmonella* LPS was demonstrated in earlier studies [21–23,48,49]. For instance, monoclonal IgA antibodies directed against the O:9 or H:g,m antigens of *S*. Enteritidis conferred protection by interfering with *Salmonella* attachment and penetration into epithelial cells, a mechanism that does not depend on the involvement of the antibody Fc region [49]. Alternatively, our results showed that mAb-449 engages FcRs to enhance macrophage bacterial uptake [19].

In conclusion, our study showed that mAb-449 is sufficient to confer protective immunity against *Salmonella* infection in mice and identified the LPS O-antigen as a prominent B-cell immunogen that mediates this process. In addition, we demonstrated a mechanism for antibody-mediated protection, wherein FcR–mAb-449 binding enhanced the uptake and killing of *Salmonella* by macrophages. We suggest that, although *S*. Typhimurium has the potential to survive and replicate within macrophages, host production of a specific antibody can effectively mediate macrophage activation for clearance of intracellular bacteria. Altogether, these data suggest that the continued study of mAb-449 will contribute to the understanding of antibody-mediated protection against *Salmonella* infection, which will provide a foundation for the further development of antibody-based approaches for effective vaccine design.

## Supporting Information

S1 Fig Analysis of sera from mice immunized with UF20. (A) Intracellular *S*. Typhimurium bacteria were quantified following infection for 1 h at MOI 5 of bacteria treated with 1% immune serum for 30 min at 37°C using Raw264.7 cells. (B) Female BALB/c mice were intravenously inoculated with UF20 or immune serum before *S*. Typhimurium challenge (n = 4). The Kaplan-Meier log-rank test stratified by regimen was significant (*p* < 0.008). (C) RAW264.7 cells were infected with *S*. Typhimurium (MOI 5), and intracellular bacteria were enumerated by culturing in LB medium. (D) mAb-449 antibody subtyping by ELISA. (A, C, D) Graphs show means and SD of triplicate readings. Significance was assessed using Student’s t test.
(TIFF)

S2 Fig Increased adherence of mAb-449-treated *S*. Typhimurium bacteria to RAW264.7 cells. (A–F) The number of adherent *S*. Typhimurium bacteria was quantified after pre-treatment with mAb-449, control IgG, or No-antibody (PBS). (A) *S*. Typhimurium (MOI 1) were treated with 0.1, 0.5, 1, 5, or 10 μg/mL mAb-449, control IgG, or PBS. Two-way ANOVA analysis showed significant differences in bacterial adherence with increased mAb-449 concentration (*p* < 0.0001). (B) MOI 0.1, (C) MOI 0.5, (D) MOI 1, (E) MOI 5, and (F) MOI 10 *S*. Typhimurium were treated with 5 μg/mL mAb-449 or control IgG before infection. (G) J774.1 mouse macrophage-like cells and (H) peritoneal macrophages were infected with *S*. Typhimurium (MOI 1) after treatment with 5 μg/mL mAb-449 or control IgG. One hour after infection, intracellular bacteria were quantified by serial dilution plating on LB. (A-H) Graphs show means and SD of triplicate readings. (B-H) Significance was assessed using Student’s t test.
(TIFF)

S3 Fig Production of nitric oxide evaluated by Griess assay after infection of RAW264.7 cells and in the presence of an NOS inhibitor. The culture supernatants were assayed for nitrite at (A) 24 h, (B) 36 h and (C) 72 h of infection. N.D., not detected. Significance was assessed using Student’s t test. Asterisks indicate statistical significance when compared to no-inhibitor group (*P*<0.05).
(TIFF)

S4 Fig Localization of *S*. Typhimurium in RAW264.7 cells. Image of RAW264.7 cells 36 h after infection with *S*. Typhimurium (green). Bar = 25 μm.
(TIFF)

S5 Fig Bacterial number measured as CFUs. RAW264.7 cells were infected with pre-treated *S*. Typhimurium (MOI 1) and the number of intracellular bacteria was determined at 36 h.
(TIFF)

S6 Fig Infection of RAW264.7 cells with similar numbers of intracellular bacteria with CFU as mAb-449-treated *S*. Typhimurium. (A) Number of intracellular bacteria in CFUs. (B) Relative CFU (%) of intracellular *S*. Typhimurium 36 h after infection in RAW264.7 cells compared to samples taken 1 h post infection. Significance was assessed using Student’s t test. Asterisks indicate statistical significance when compared to 1h group (*P* = 0.0483).
(TIFF)

S7 Fig Nitric oxide production evaluated by Griess assay after 36 h of infection. RAW264.7 cells were infected with mAb-449-treated *S*. Typhimurium MOI 1 and control IgG-treated MOI 10. Significance was assessed using Student’s t test.
(TIFF)

S8 Fig Determination of binding affinities of LPS from *S*. Typhimurium with mAb-449. ELISA analysis showing antibody-antigen recognition with mAb-449 and LPS. Significance was assessed using Student’s t test.
(TIFF)

S9 Fig Effect of mAb-449 on intracellular bacterial killing after treatment with NOS inhibitor. Relative CFU (%) of intracellular *S*. Typhimurium 36 h after infection in RAW264.7 cells compared to samples taken 1 h post infection. Significance was assessed using Student’s t test. * *P* = 0.0124.
(TIFF)

S1 Protocol (PDF)
