# Supplementary figures and images for: Specific Monoclonal Antibody Overcomes the Salmonella enterica Serovar Typhimurium’s Adaptive Mechanisms of Intramacrophage Survival and Replication
Source: PLoS One. 2016 Mar 17;11(3):e0151352. doi: 10.1371/journal.pone.0151352 (PMC4795626; doi:10.1371/journal.pone.0151352)

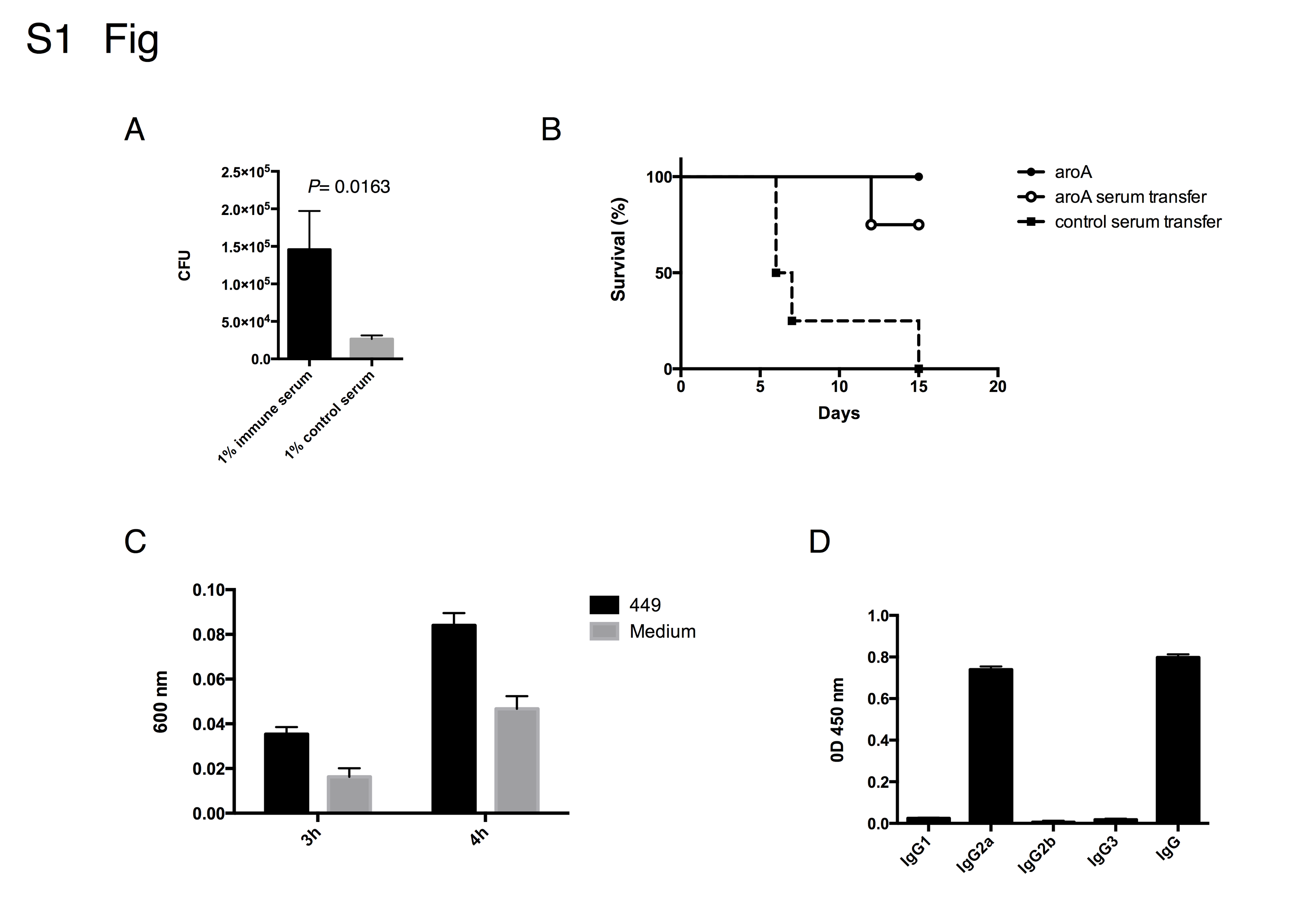

Supplement: S1 Fig — (A) Intracellular S. Typhimurium bacteria were quantified following infection for 1 h at MOI 5 of bacteria treated with 1% immune serum for 30 min at 37°C using Raw264.7 cells. (B) Female BALB/c mice were intravenously inoculated with UF20 or immune serum before S. Typhimurium challenge (n = 4). The Kaplan-Meier log-rank test stratified by regimen was significant (p < 0.008). (C) RAW264.7 cells were infected with S. Typhimurium (MOI 5), and intracellular bacteria were enumerated by culturing in LB medium. (D) mAb-449 antibody subtyping by ELISA. (A, C, D) Graphs show means and SD of triplicate readings. Significance was assessed using Student’s t test. (TIFF) [file pone.0151352.s001.tiff]

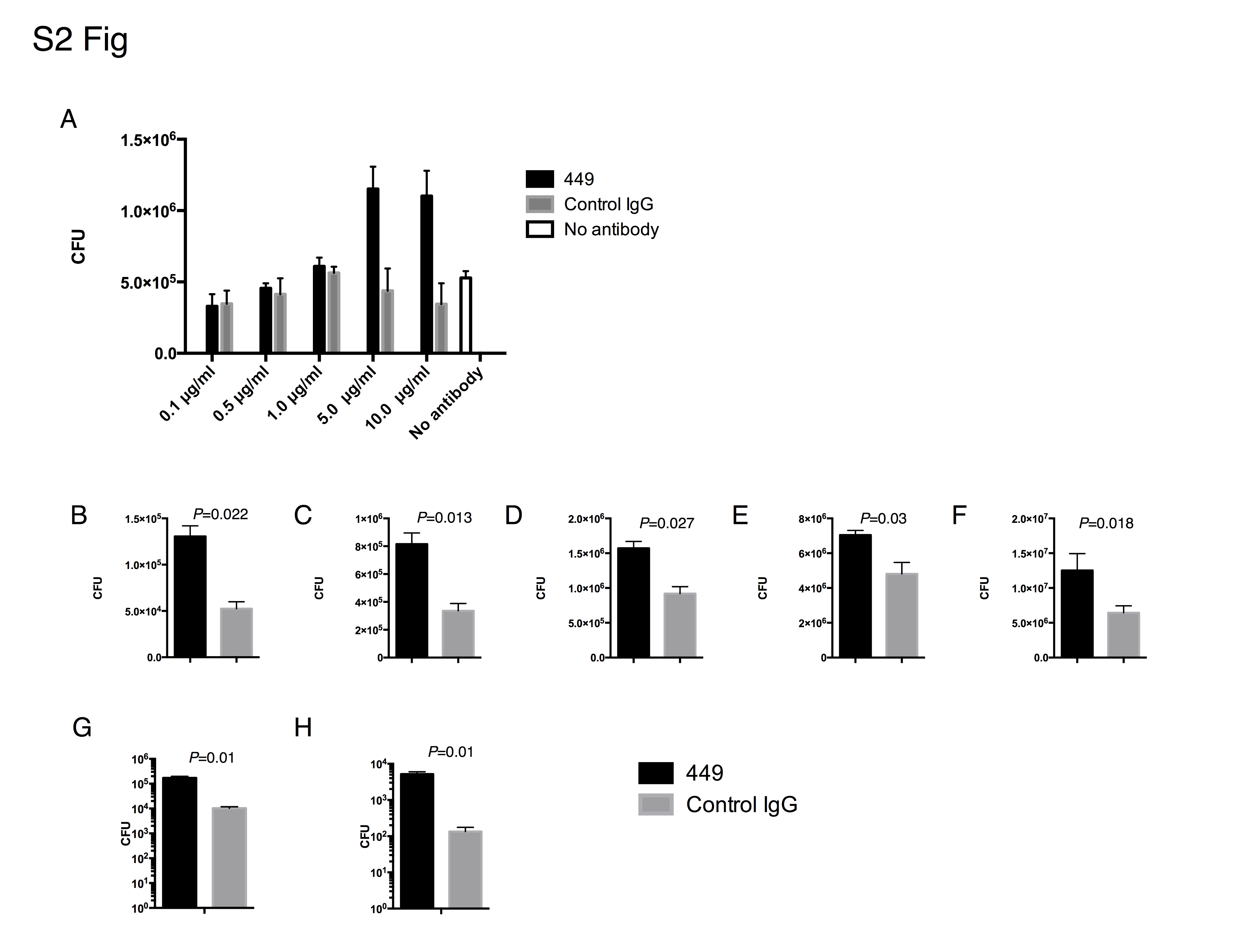

Supplement: S2 Fig — (A–F) The number of adherent S. Typhimurium bacteria was quantified after pre-treatment with mAb-449, control IgG, or No-antibody (PBS). (A) S. Typhimurium (MOI 1) were treated with 0.1, 0.5, 1, 5, or 10 μg/mL mAb-449, control IgG, or PBS. Two-way ANOVA analysis showed significant differences in bacterial adherence with increased mAb-449 concentration (p < 0.0001). (B) MOI 0.1, (C) MOI 0.5, (D) MOI 1, (E) MOI 5, and (F) MOI 10 S. Typhimurium were treated with 5 μg/mL mAb-449 or control IgG before infection. (G) J774.1 mouse macrophage-like cells and (H) peritoneal macrophages were infected with S. Typhimurium (MOI 1) after treatment with 5 μg/mL mAb-449 or control IgG. One hour after infection, intracellular bacteria were quantified by serial dilution plating on LB. (A-H) Graphs show means and SD of triplicate readings. (B-H) Significance was assessed using Student’s t test. (TIFF) [file pone.0151352.s002.tiff]

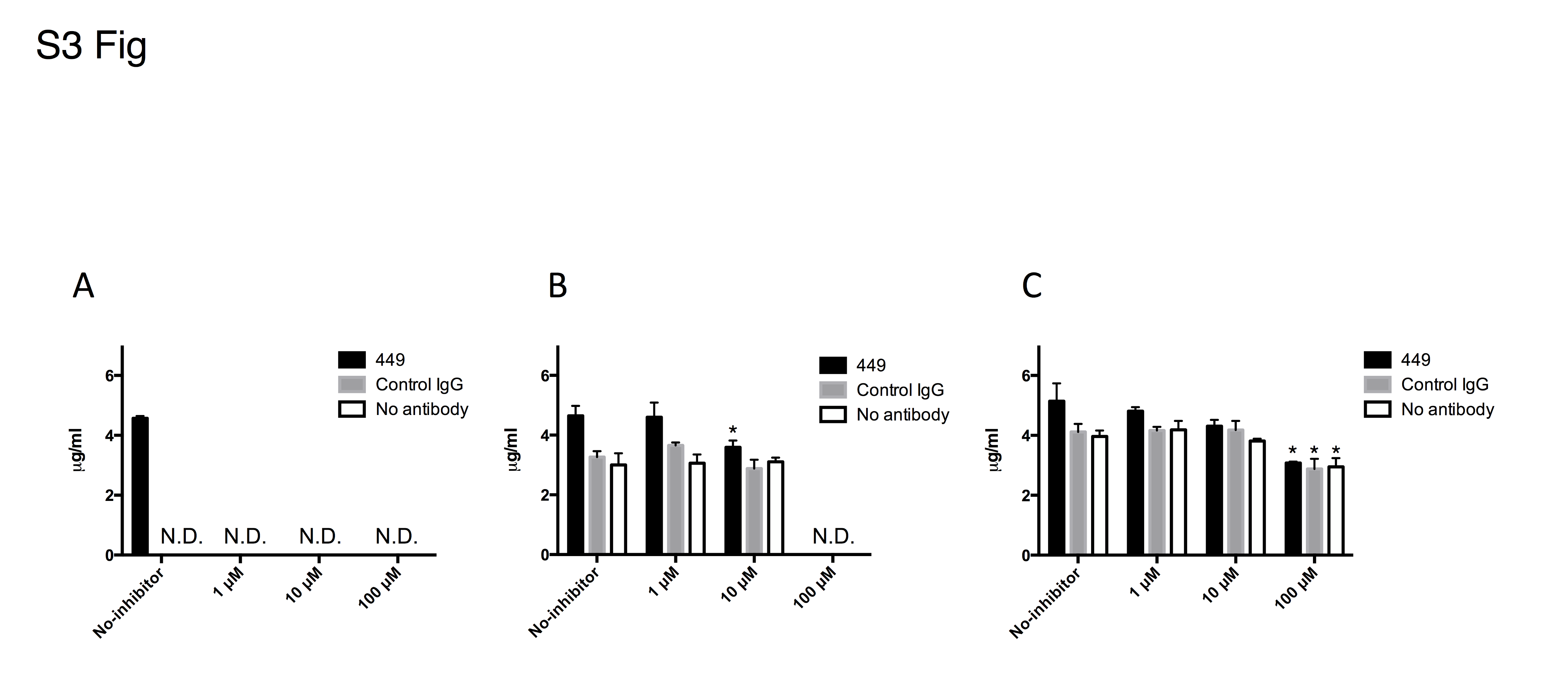

Supplement: S3 Fig — The culture supernatants were assayed for nitrite at (A) 24 h, (B) 36 h and (C) 72 h of infection. N.D., not detected. Significance was assessed using Student’s t test. Asterisks indicate statistical significance when compared to no-inhibitor group (P<0.05). (TIFF) [file pone.0151352.s003.tiff]

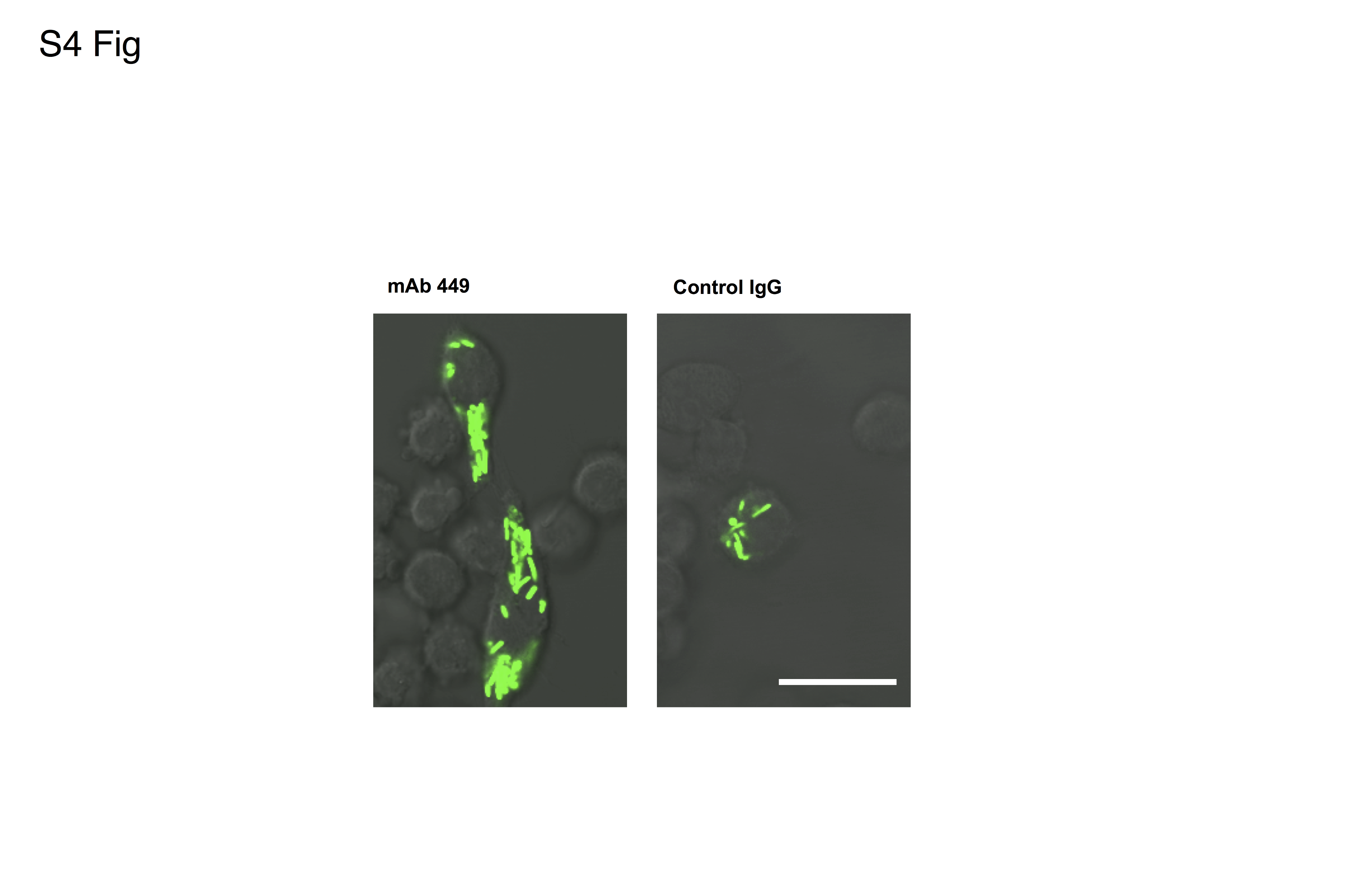

Supplement: S4 Fig — Image of RAW264.7 cells 36 h after infection with S. Typhimurium (green). Bar = 25 μm. (TIFF) [file pone.0151352.s004.tiff]

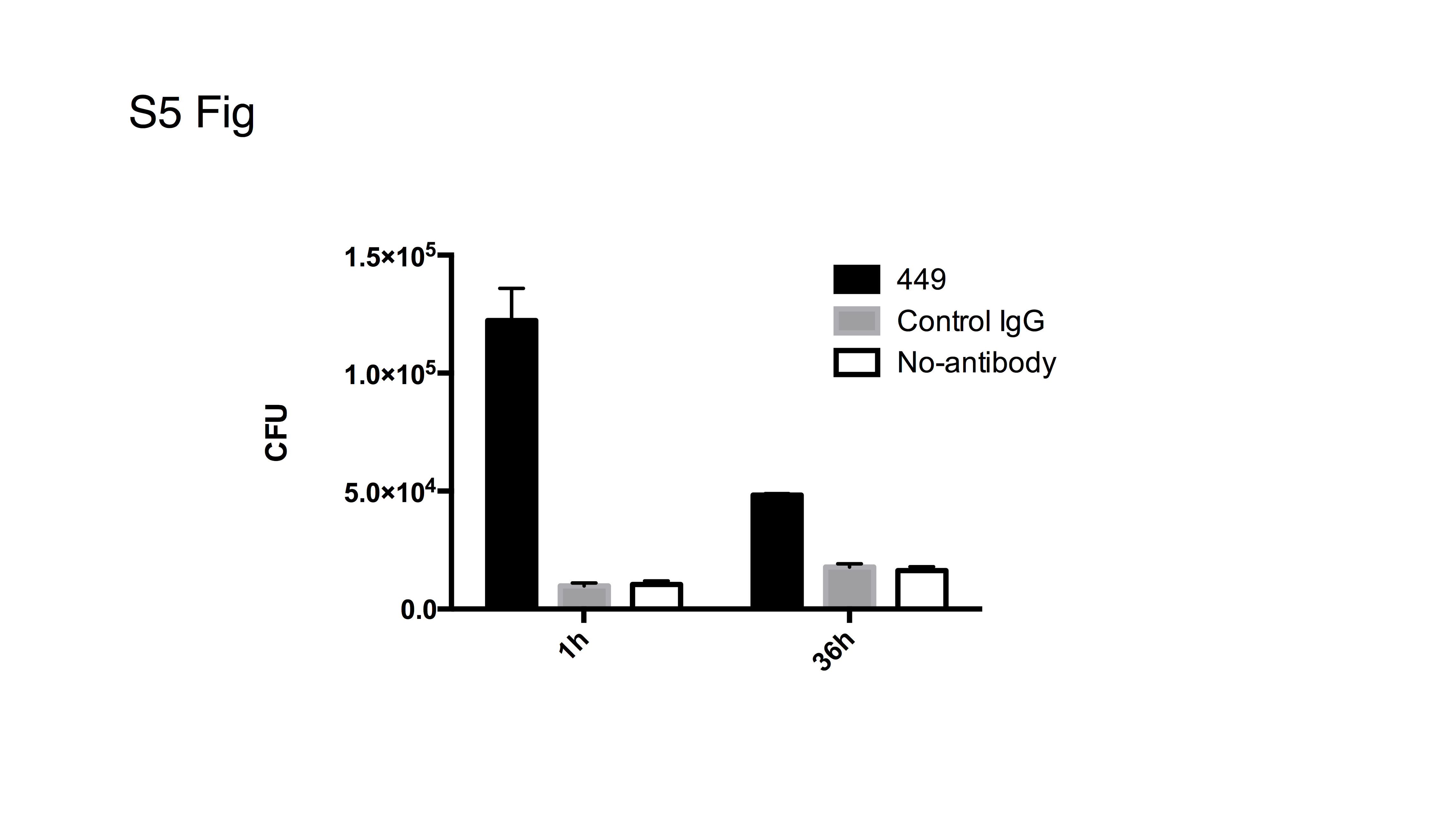

Supplement: S5 Fig — RAW264.7 cells were infected with pre-treated S. Typhimurium (MOI 1) and the number of intracellular bacteria was determined at 36 h. (TIFF) [file pone.0151352.s005.tiff]

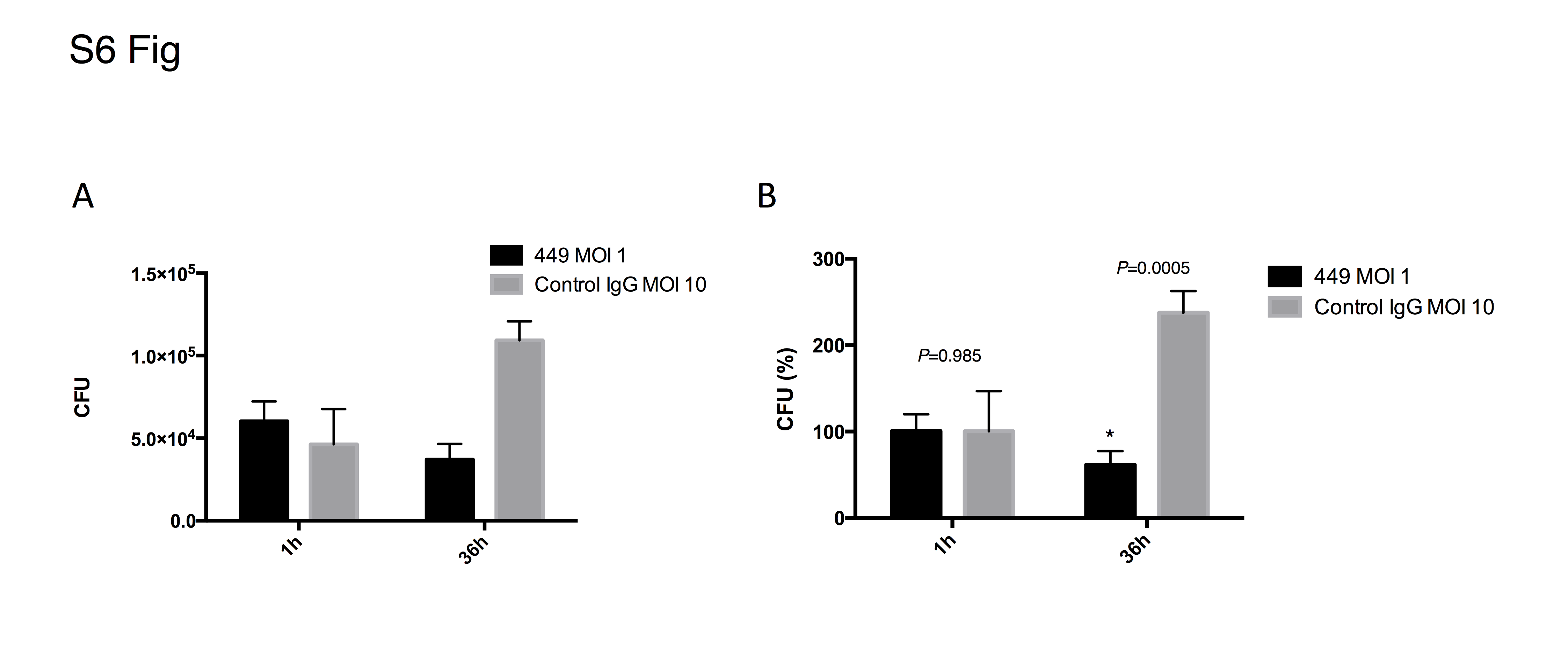

Supplement: S6 Fig — (A) Number of intracellular bacteria in CFUs. (B) Relative CFU (%) of intracellular S. Typhimurium 36 h after infection in RAW264.7 cells compared to samples taken 1 h post infection. Significance was assessed using Student’s t test. Asterisks indicate statistical significance when compared to 1h group (P = 0.0483). (TIFF) [file pone.0151352.s006.tiff]

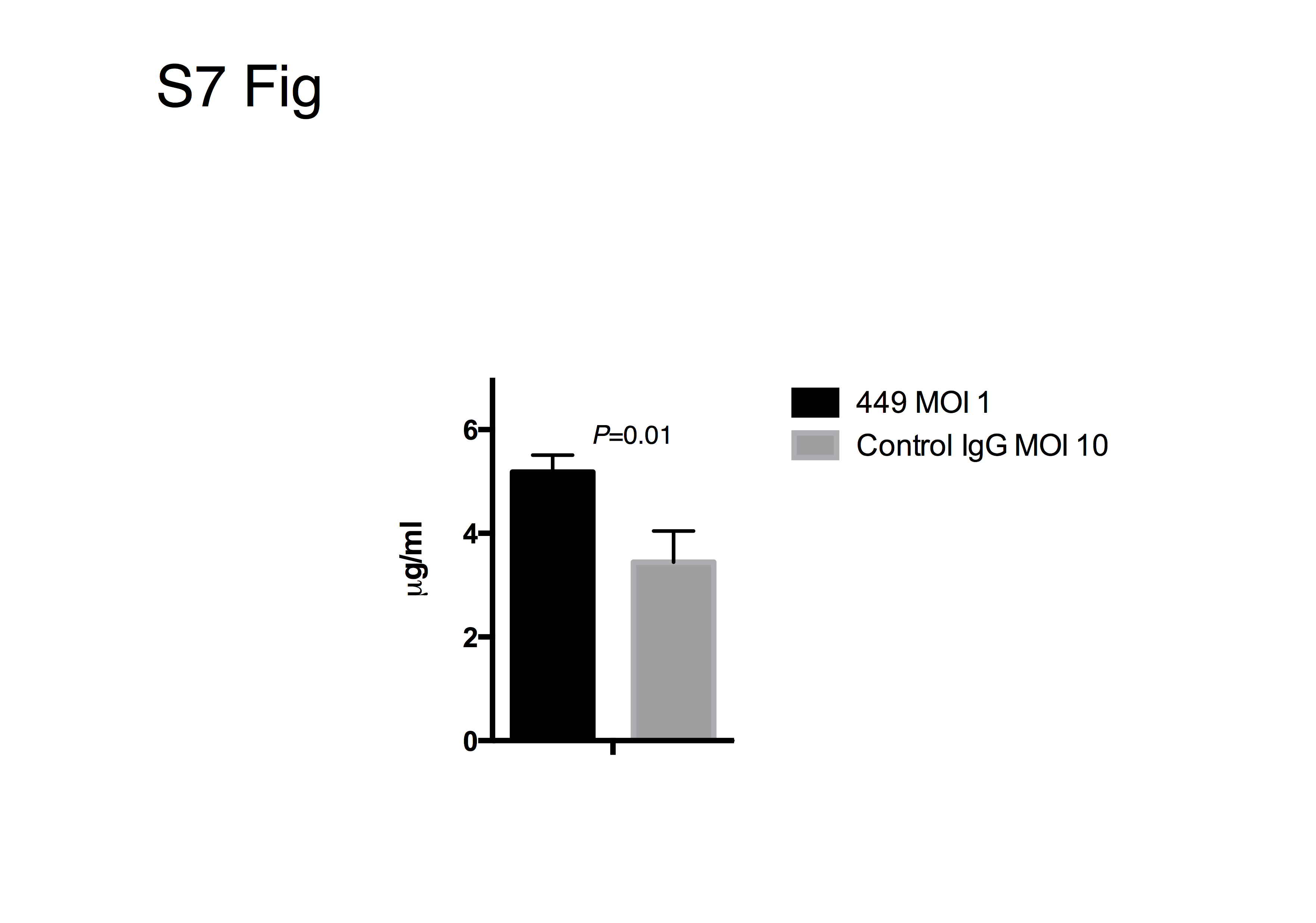

Supplement: S7 Fig — RAW264.7 cells were infected with mAb-449-treated S. Typhimurium MOI 1 and control IgG-treated MOI 10. Significance was assessed using Student’s t test. (TIFF) [file pone.0151352.s007.tiff]

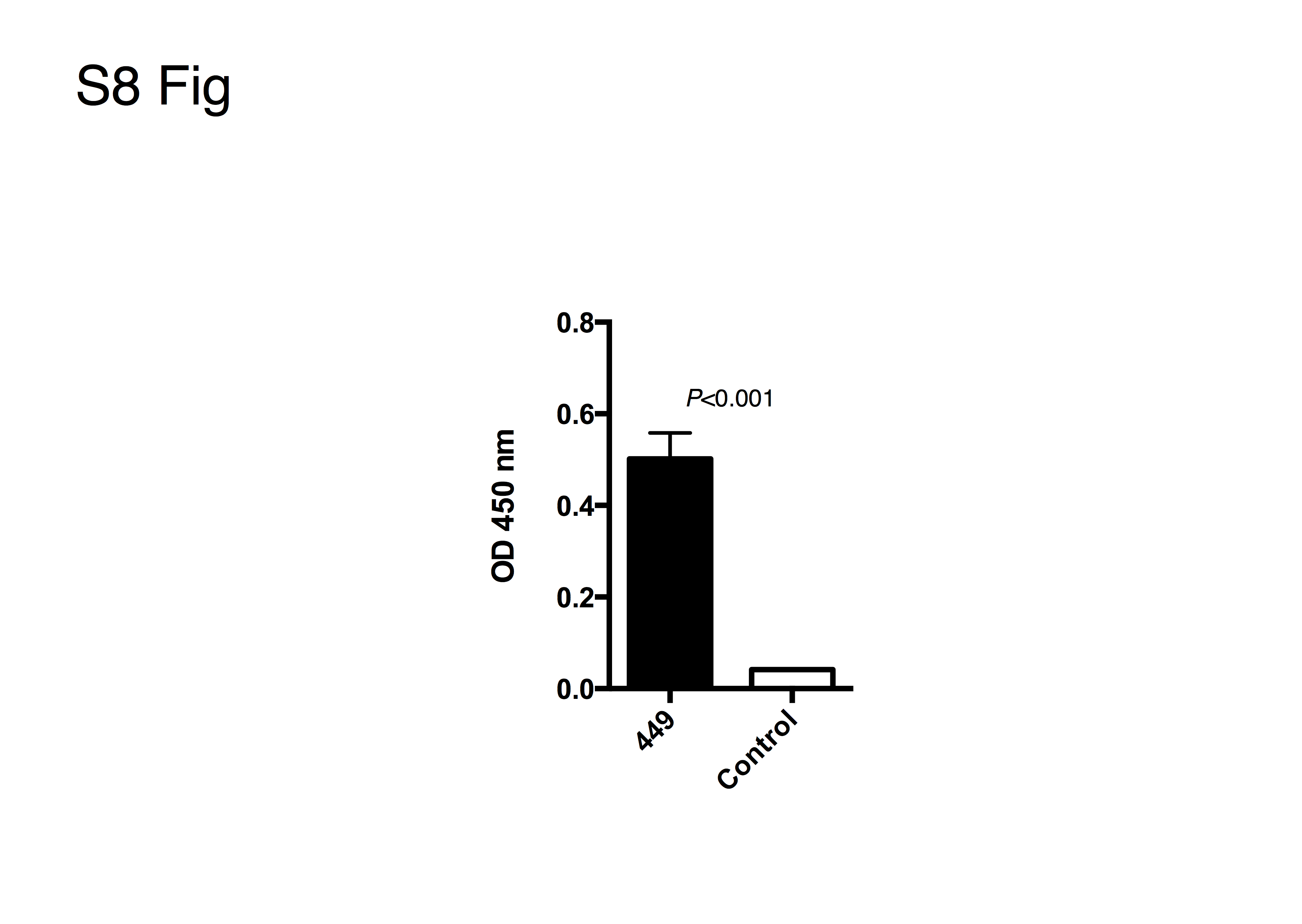

Supplement: S8 Fig — ELISA analysis showing antibody-antigen recognition with mAb-449 and LPS. Significance was assessed using Student’s t test. (TIFF) [file pone.0151352.s008.tiff]

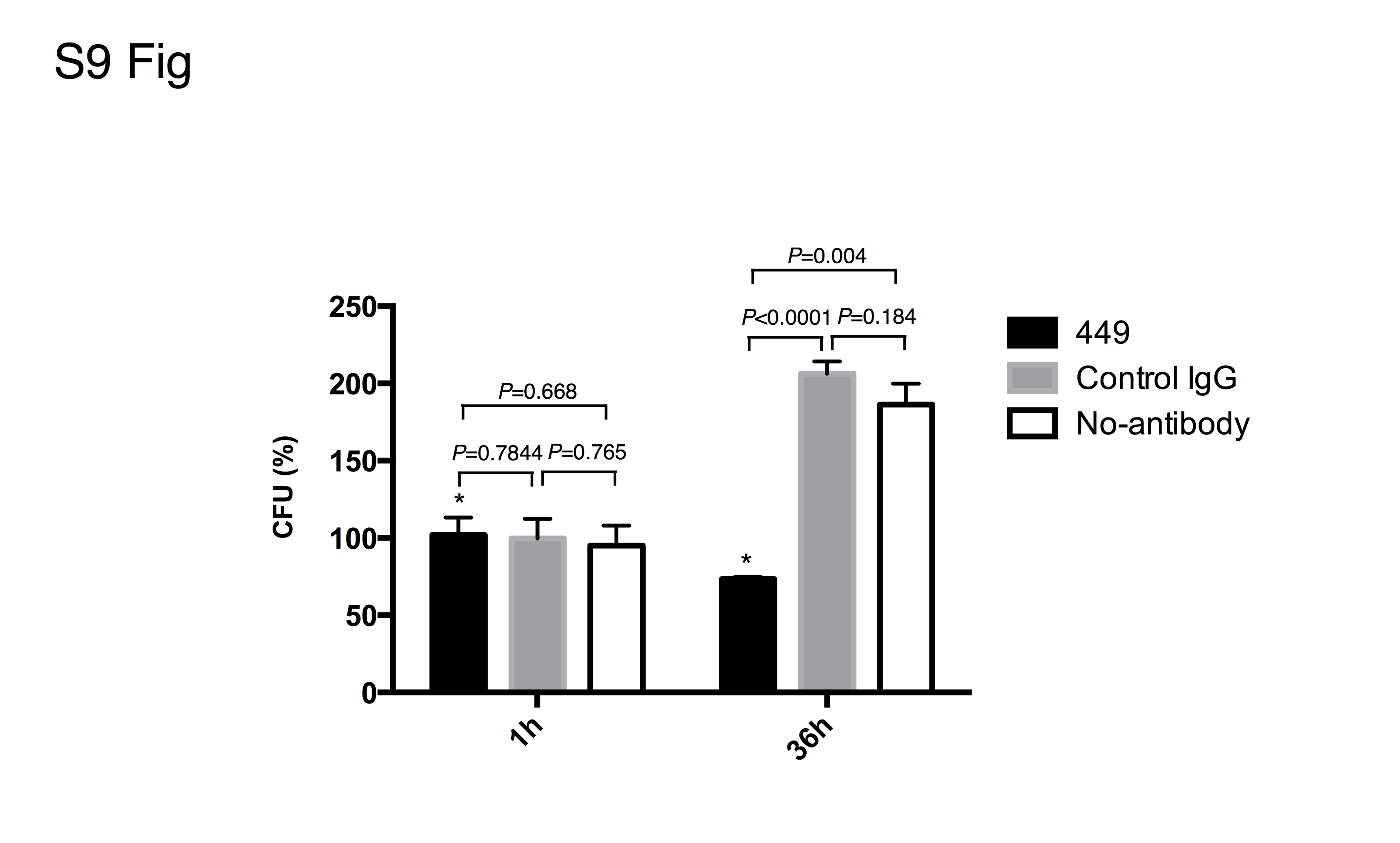

Supplement: S9 Fig — Relative CFU (%) of intracellular S. Typhimurium 36 h after infection in RAW264.7 cells compared to samples taken 1 h post infection. Significance was assessed using Student’s t test. * P = 0.0124. (TIFF) [file pone.0151352.s009.tiff]
