# Supplementary material for: Specific Monoclonal Antibody Overcomes the Salmonella enterica Serovar Typhimurium’s Adaptive Mechanisms of Intramacrophage Survival and Replication
Source: PLoS One. 2016 Mar 17;11(3):e0151352. doi: 10.1371/journal.pone.0151352 (PMC4795626; doi:10.1371/journal.pone.0151352)
Supplement: S1 Protocol — (PDF) [file pone.0151352.s010.pdf]

# Supporting Information

# S1 Protocol.

## Generation of the mAb-449-producing hybridoma

The mAb-449 hybridoma was obtained by infecting 6-week-old BALB/c mice intravenously with $5 \times 10^5$ CFUs *Salmonella enterica* serovar Typhimurium UF20 (*aroA*$^-$) as previously described [1,2] . Spleens were harvested at 60 days post-infection, and isolated splenocytes were fused with P3-X63-Ag8-U1 myeloma cells using 50% polyethylene glycol (Hampton Research, Aliso Viejo, CA, USA) [3]. Hybridoma cells were cultured in hypoxanthine-aminopterin-thymidine (HAT) selection medium for 7 days, and the medium was assayed for specificity using a modified in vitro infection assay [4].

## Enzyme-linked immunosorbent assay (ELISA)

For ELISA studies, wells coated with 5 μg/mL IgG1, 2a, 2b, and 3 (Invitrogen, Carlsbad, CA, USA) or LPS from S. Typhimurium were incubated in the

hybridoma cell medium or mAb 449 (2 μg/mL). Bound antibodies were then detected with HRP-conjugated rabbit anti-mouse IgG1, 2a, 2b, and 3 (Invitrogen, Carlsbad, CA, USA) as secondary antibodies and developed using 3,3',5,5'-tetramethylbenzidine (TMB) substrate solution (Kirkegaard & Perry Laboratories, Inc., Gaithersburg, MD, USA). The reaction was stopped by adding 100 μL 1 M phosphoric acid to each well [5], and the absorbance was read at 450 nm using a microplate spectrophotometer (Perkin Elmer, Waltham, MA, USA).

## Analysis of mAb-449 by a modified in vitro infection study

RAW264.7 cells at a density of 1 × $10^5$ cells/well were infected with *S.* Typhimurium treated with mAb-449-producing hybridoma medium for 1 h at 37 °C. After washing with PBS, the cells were lysed with 30 μL 0.2% Triton X-100 in PBS, and 100 μL LB broth was added as previously described [4].

Bacterial growth was measured with a microplate reader at 600 nm at different time points.

## Macrophage experiments

For macrophage function assays, mouse macrophage-like RAW264.7, J774.1, or peritoneal macrophages prepared from naïve BALB/c mice were plated at 1 × $10^5$ cells/well and infected with MOI 1 of *S.* Typhimurium pre-treated with 5 µg/mL mAb-449 or control IgG for 30 min at 37 °C. After 1 h of infection, macrophages were washed with PBS. The infected macrophages were then lysed in 1 mL 0.2% Triton X-100 in PBS for 5 min to enumerate intracellular bacteria on LB plates.

## The Nitric oxide synthesis inhibitor.

Raw264.7 cells were infected as described in Materials and Methods. The inhibitor $N^G$-Monomethyl-L-arginine, acetate (L-NMMA) (DOJINDO, Kumamoto, Japan) was added with 30 µg/mL gentamicin after 1h of 100 µg/mL gentamicin

treatment [6]. Nitric oxide production assay were performed as described in Materials and Methods.

## Confocal scanning laser microscope.

Green fluorescent protein (GFP)-labeled *S.* Typhimurium X3306 was used in this study [2]. Raw264.7 cells were infected as described in Materials and Methods. The infected Raw264.7 cells were examined with a Leica TCS-SP5 confocal scanning laser microscope for detection of bacteria within Raw264.7 cells.

# References


1. Gulig PA, Doyle TJ (1993) The *Salmonella typhimurium* virulence plasmid increases the growth rate of salmonellae in mice. Infect Immun 61: 504-511.
2. Eguchi M, Kikuchi Y (2010) Binding of *Salmonella*-specific antibody facilitates specific T cell responses via augmentation of bacterial uptake and induction of apoptosis in macrophages. J Infect Dis 201: 62-70.
3. Aribam SD, Ogawa Y, Matsui H, Hirota J, Okamura M, et al. (2015) Monoclonal antibody-based competitive enzyme-linked immunosorbent

assay to detect antibodies to O:4 *Salmonella* in the sera of livestock and poultry. J Microbiol Methods 108: 1-3.

4. Aribam SD, Hirota J, Kusumoto M, Harada T, Shiraiwa K, et al. (2014) A rapid differentiation method for enteroinvasive *Escherichia coli*. J Microbiol Methods 98: 64-66.

5. Ko HJ, Yang JY, Shim DH, Yang H, Park SM, et al. (2009) Innate immunity mediated by MyD88 signal is not essential for induction of lipopolysaccharide-specific B cell responses but is indispensable for protection against *Salmonella enterica* serovar Typhimurium infection. J Immunol 182: 2305-2312.

6. Chakravortty D, Hansen-Wester I, Hensel M (2002) *Salmonella* pathogenicity island 2 mediates protection of intracellular *Salmonella* from reactive nitrogen intermediates. J Exp Med 195: 1155-1166.
